# Zika virus transmission to mouse ear by mosquito bite: a laboratory model that replicates the natural transmission process

**DOI:** 10.1186/s13071-017-2286-2

**Published:** 2017-07-20

**Authors:** Nagila Francinete Costa Secundino, Barbara Aparecida Chaves, Alessandra Silva Orfano, Karine Renata Dias Silveira, Nilton Barnabe Rodrigues, Thais Bonifácio Campolina, Rafael Nacif-Pimenta, Luiz Eduardo Martinez Villegas, Breno Melo Silva, Marcus Vinícius Guimarães Lacerda, Douglas Eric Norris, Paulo Filemon Paolucci Pimenta

**Affiliations:** 1 Laboratory of Medical Entomology, René Rachou Research Centre – FIOCRUZ-MG, Belo Horizonte, Minas Gerais Brazil; 2 Tropical Medicine Foundation Dr. Heitor Vieira Dourado, Manaus, Amazonas Brazil; 3 Department of Biological Sciences, Federal University of Ouro Preto, Ouro Preto, Minas Gerais Brazil; 4 Leonidas e Maria Deane Research Centre – FIOCRUZ, Manaus, Amazonas Brazil; 5 Department of Molecular Microbiology and Immunology, Johns Hopkins Bloomberg School of Public Health, Baltimore, USA

**Keywords:** Transmission, Laboratory model, Zika virus, *Aedes aegypti*

## Abstract

**Background:**

Zika disease has transformed into a serious global health problem due to the rapid spread of the arbovirus and alarming severity including congenital complications, microcephaly and Guillain-Barré syndrome. Zika virus (ZIKV) is primarily transmitted to humans through the bite of an infective mosquito, with *Aedes aegypti* being the main vector.

**Methods:**

We successfully developed a ZIKV experimental transmission model by single infectious *Ae*. *aegypti* bite to a laboratory mouse using circulating Brazilian strains of both arbovirus and vector. Mosquitoes were orally infected and single *Ae*. *aegypti* were allowed to feed on mouse ears 14 days post-infection. Additionally, salivary gland (SG) homogenates from infected mosquitoes were intrathoracically inoculated into naïve *Ae*. *aegypti*. Mosquito and mouse tissue samples were cultured in C6/36 cells and processed by quantitative real-time PCR.

**Results:**

A total of 26 *Ae*. *aegypti* were allowed to feed individually on mouse ears. Of these, 17 mosquitoes fed, all to full engorgement. The transmission rate of ZIKV by bite from these engorged mosquitoes to mouse ears was 100%. The amount of virus inoculated into the ears by bites ranged from 2 × 10^2^–2.1 × 10^10^ ZIKV cDNA copies and was positively correlated with ZIKV cDNA quantified from SGs dissected from mosquitoes post-feeding. Replicating ZIKV was confirmed in macerated SGs (2.45 × 10^7^ cDNA copies), mouse ear tissue (1.15 × 10^3^ cDNA copies, and mosquitoes 14 days post-intrathoracic inoculation (1.49 × 10^7^ cDNA copies) by cytopathic effect in C6/36 cell culture and qPCR.

**Conclusions:**

Our model illustrates successful transmission of ZIKV by an infectious mosquito bite to a live vertebrate host. This approach offers a comprehensive tool for evaluating the development of infection in and transmission from mosquitoes, and the vertebrate-ZIKV interaction and progression of infection following a natural transmission process.

## Background

Zika disease has transformed into a serious global health problem due to the rapid spread of the arbovirus and alarming severity including congenital complications, microcephaly and Guillain-Barré syndrome [1]. Zika virus (ZIKV) is primarily transmitted to humans through the bites of infective mosquitoes. *Aedes aegypti* is considered to be the main mosquito vector, is widespread in the Americas, and is additionally responsible for transmission of several other epidemiologically important arboviruses including dengue and chikungunya [2]. *Aedes aegypti* mosquitoes have a preference for feeding on humans, oviposit in artificial and natural containers, and therefore thrive in urban environments around the globe where they have been inadvertently introduced [3]. Sociological, ecological and epidemiological conditions in many heavily populated cities, including those in Brazil at the center of the ZIKV epidemic, are permissive for *Ae*. *aegypti* endemicity and, therefore, for invasion and circulation of ZIKV.

The transmission of arboviruses to vertebrates by mosquito vectors is complex and requires an in-depth understanding of vector-pathogen interaction and biological transmission, which involves three essential organisms: vector, virus and vertebrate. Animal models using rhesus macaques, mice and hamsters have been proposed for understanding ZIKV infection, development and pathogenesis in vertebrates. These animal models are experimentally infected by syringe inoculation of cultivated virus [4–9]. However, this bypasses natural transmission mechanisms and virus inoculum introduced by mosquito bite. Furthermore, it is uncertain if infection initiated by syringe inoculation adequately mimics Zika disease progression and pathogenesis following natural transmission by mosquito bite. It is well documented in other models that syringe-inoculation of cultured vector-borne pathogen results in physiological conditions of the vertebrate host response that are altered, compared to natural vector transmission [10–12]. The transmission of pathogens by vector bite is thought to influence and facilitate pathogen invasion and replication by providing a variety of vascular-dilatators, anti-hemostatic and immune-modulatory substances in the saliva [13]. Inarguably, establishing vector transmission by bite is the most epidemiologically relevant mode to study the infectious process of ZIKV transmission.

The Zika outbreak in South America raised global alarm with the revelation that ZIKV pathogenesis in humans could cause severe conditions such as miscarriage and microcephaly, in addition to Guillain-Barré syndrome that had already been recognized from the Pacific island outbreaks. The mechanisms of these viral-induced pathologies remain poorly understood. After an infective bite, the first barrier to the virus is the human skin where ZIKV induces antiviral immune responses. Hamel et al. [14] demonstrated that human dermal cells such as fibroblasts, keratinocytes and dendritic cells are permissive to the virus. Additionally, virus replication activates an antiviral innate immune response by the host, with production of interferon by the infected cells. To most appropriately explore pathogenesis in vertebrates, an animal model that replicates natural transmission of ZIKV *via* mosquito vector is immediately required.

Here, we have successfully developed an experimental ZIKV transmission model by a single ZIKV-infectious *Ae*. *aegypti* bite to a laboratory mouse using circulating Brazilian strains of both arbovirus and vector. In contrast with published procedures for arboviruses [15–17], this approach offers a novel tool for evaluating the development of ZIKV infection following a natural transmission process.

## Methods

### Mosquito rearing

A well-established Brazilian colony of *Ae*. *aegypti* (strain PP-Campos), maintained at the Laboratory of Medical Entomology, Fiocruz-MG, Brazil, was used in this study. Mosquito eggs were collected using ovitraps in the city of Campos dos Goitacazes, State of Rio de Janeiro, Brazil, in 2000. Mosquitoes were reared and maintained under standard insectary conditions (27 °C, 80% relative humidity, 16 h light/8 h dark photoperiod).

### Zika virus

A currently circulating Brazilian human isolate of ZIKV from the State of São Paulo (ZIKV/*H*. *sapiens*/Brazil/SPH/2015) was used in all experiments [18]. Prior to the experiments, virus stocks were passaged in an *Ae*. *albopictus* cell line (C6/36) grown in Leibowitz L-15 medium supplemented with 2% inactivated fetal bovine serum, 20 μg/ml Gentamicin, 5 μg/ml Anphotericin B, and 200 U/ml Penicillin [19]. Virus titration followed the TCID50 method [20].

### ZIKV infection of *Ae*. *aegypti*

Female *Ae*. *aegypti* (3–5 day-old) were orally infected *via* glass feeding devices filled with ZIKV-infected C6/36 cell culture supernatant re-suspended 2:1 in fresh mouse blood, as described previously [19]. A blood meal with ZIKV titer of 5 × 10^5^ PFU/ml was calculated as previously described [21] and used in all infection experiments. Mosquitoes were allowed to feed for 30 min on the ZIKV-infective blood meal. After feeding, approximately 300 fully-engorged females were separated into new cages and maintained on 10% glucose solution ad libitum for up to 14 days post-infection (dpi). To determine blood meal size and estimate the number of ZIKV viral copies ingested, a group of 20 mosquitoes were weighed before and after the ingestion of the infective blood meal in parallel.

### ZIKV transmission by *Ae*. *aegpyti* bite

Fourteen days after the ZIKV-infective blood meal, *Ae*. *aegypti* mosquitoes were individually placed into small plastic vials (3 dram volume, 4.8 cm height, 1.8 cm diameter) covered at one end with a 0.25 mm nylon mesh. Each vial and a single, potentially infectious mosquito was placed with the mesh side against an ear of an anesthetized BALB/c mouse. A total of 13 mice were exposed to a total of 26 mosquitoes (one mosquito/mouse ear). The female mosquitoes were allowed to bite and feed for 30 min. Mice were immediately euthanized following mosquito bite exposure and the mosquito-exposed region of each ear biopsied with a 4 mm punch. Subsequently, all fully engorged mosquitoes were killed quickly by cold exposure, removed from the vials, and bodies and salivary glands (SGs) dissected. Three sample types resulted from this experiment: (i) BALB/c mouse ear punch biopsies; (ii) post-bite *Ae*. *aegypti* bodies; and (iii) *Ae*. *aegypti* salivary glands.

### Viability of ZIKV transmitted by *Ae*. *aegypti* bite

Supernatants of each dissected post-bite SG (group iii) were divided in two equal parts and processed for: (a) cultivation in C6/36 cells for 5 days as described above for observation of ZIKV growth and cytopathic effect [22]; and (b) C6/36 supernatants were processed for qPCR for ZIKV cDNA copy quantification.

### Intrathroracic inoculation for confirmation of salivary gland-ZIKV infectivity

In parallel to the bite/transmission experiments, 14 days after the ZIKV-infective blood meal, 10 randomly selected *Ae*. *aegypti* mosquitoes were killed quickly by cold exposure, SGs dissected, and macerated SGs used for intrathoracic inoculations of 20 naïve 3 to 5 day old *Ae*. *aegypti* (Nanoinjector II, Drummond Scientific Co., Broomal, USA). These mosquitoes were maintained post-inoculation on 10% glucose solution ad libitum for 14 days and processed by qPCR for ZIKV quantification to verify that ZIKV in the originally infected mosquitoes was both infectious and replication competent [23].

### Viability of ZIKV introduced into mouse ears by mosquito bite

In parallel, 5 mice were euthanized immediately after being bitten by ZIKV infected *Ae*. *aegypti*. The ears were removed, washed in 70% ethanol and PBS, separated in two dermal sheets (internal and external side) and incubated at 37 °C for 90 min in 1 ml of DMEM with 40 mM Hepes and 0.2 mg/ml Liberase Enzyme blends (Roche Holding, Basel, Switzerland). Digested ear sheets were homogenized for 3 min using the Medicon/Medimachine tissue homogenizer system (Becton Dickinson, Franklin Lakes, USA). Each homogenate (single cell suspensions of tissue homogenates were) were removed with a syringe while adding 10 ml of DMEM and filtered through a 70 μm-pore size cell strainer (BD Biosciences, San Jose, CA), filtrate spun for 10 min at 1500 rpm, re-suspended in 2 ml Leibowitz L-15 medium and serially diluted into a 24-well flat bottom microtiter plate coated with C6/36 cells. The ear homogenates were cultivated for 4 days to observe growth and cytopathic effects. The supernatants were removed and processed by qPCR to estimate viral ZIKV cDNA copies, and the infected C6/36 monolayers fixed for at least 1 h with formaldehyde 10%. The cytophatic effect of the ZIKV on the cells was detected by direct visualization of plaques on the monolayers after crystal violet staining.

### Real-time qPCR for ZIKV detection and quantification

Tissues derived from all experiments, including mosquito bodies, SGs, biopsied mouse ears and homogenate subsamples were processed for RNA extraction (QIAamp viral RNA Mini kit, Qiagen, Hilden, Germany). Detection and quantification of ZIKV cDNA copies was determined by TaqMan qPCR (Thermo Fisher Scientific, Hampton, USA) as described by Lanciotti et al. [24].

### Statistical analysis

Infection rate (IR), SG infection rate (SG-IR), transmission rate (TR) and vectorial capacity (VC) were evaluated using two-tailed Chi-square or Fisher’s exact tests. Spearman’s non-parametric correlation test was used to test for a statistically significant correlation with *P* values ≤ 0.05 considered significant. All statistical analyses were performed in GraphPad Prism, version 6.00 (San Diego, CA, USA).

## Results

### Evaluation of ZIKV oral infection in *Ae*. *aegypti*

**Fig. 1:**
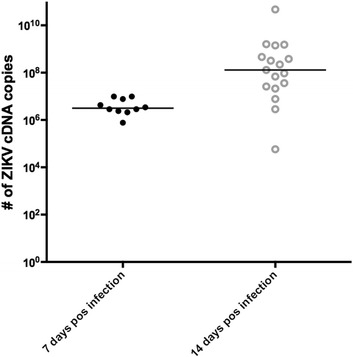
ZIKV oral infection of *Aedes aegpyti*. Number of ZIKV cDNA copies of infected mosquitoes at 7 and 14 days after the infective blood meal

*Aedes aegypti* mosquitoes were successfully infected with ZIKV after the ingestion of the infective blood meal. The mean amount of blood ingested by fully engorged mosquitoes was 3.0 μl with a range of 1.7–6.0 μl and an average ingestion of 10^2^ ZIKV cDNA copies. At 7 dpi, the infection rate was 100% (10/10), with the median of 3.1 × 10^6^ ZIKV cDNA copies/mosquito. At 14 dpi, the infection rate was 100% (17/17), with a median of 1.3 × 10^8^ ZIKV cDNA copies/mosquito. 14 dpi ZIKV-infected *Ae*. *aegypti* were used to test the virus transmission by bites to mouse ears (Fig. 1).

### ZIKV transmission by single infected *Ae*. *aegypti*

**Fig. 2:**
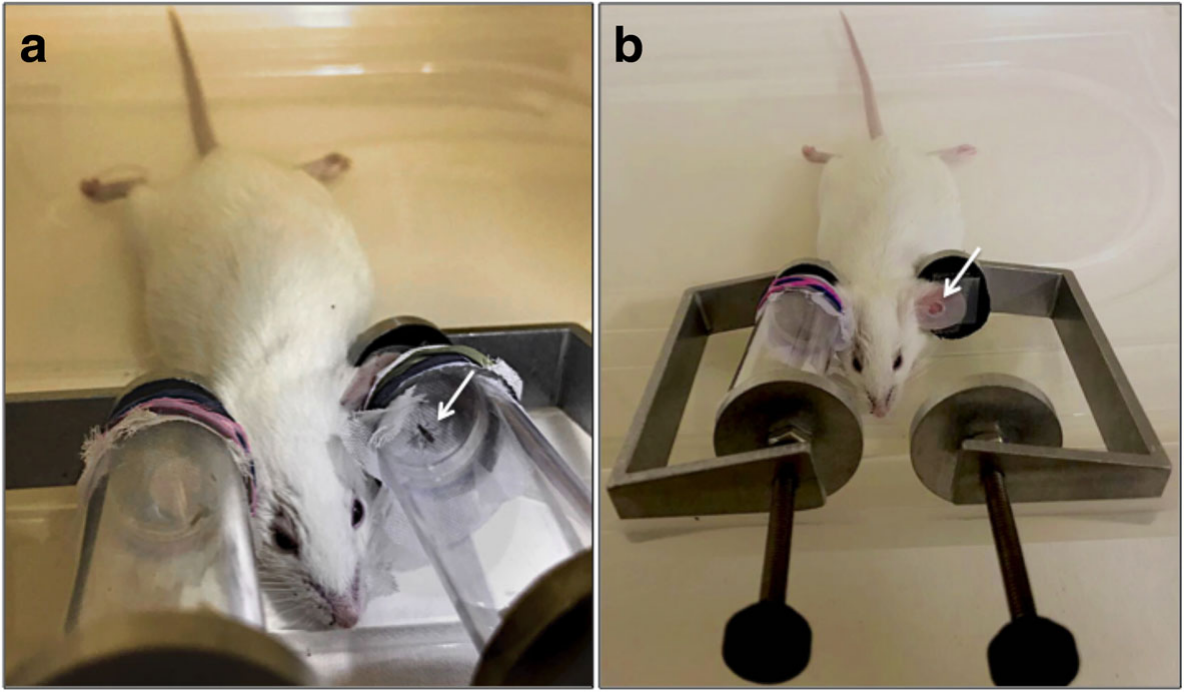
ZIKV transmission by an *Aedes aegyti* bite. In each vial a single potentially ZIKV infectious mosquito was placed with the nylon mesh side against the ears of an anesthetized BALB/c mouse. It is possible to observe a mosquito probing on the mice ear (*arrow* in **a**). **b** Mosquito-exposed region of the mice ear after removing the plastic vial (*arrow*)

**Fig. 3:**
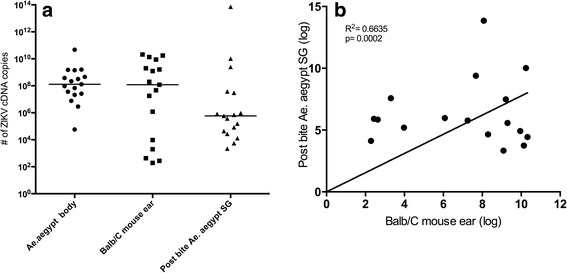
ZIKV detection by qPCR in single infected *Aegypti aegypti* and mouse ears after the transmission by bite. **a** Viral cDNA copies are plotted for mosquito bodies, mouse ears on which mosquitoes fed and salivary glands (SG) post-bite. **b** Positive correlation between cDNA copies from mouse ears and SGs dissected from mosquito that fed on each ear

Seventeen of the 26 mosquitoes placed on mouse ears for the transmission by bite experiment fed to visible engorgement (Fig. 2). The immediate dissection and analysis of these blood-fed mosquitoes revealed that they were all infected with varying amounts of ZIKV in their bodies (5.8 × 10^3^–4.7 × 10^10^cDNA copies) and SGs (2.1 × 10^4^−7.16 × 10^13^cDNA copies). The transmission rate by bite was 100%, ZIKV was detected in all 17 mouse ears from which mosquitoes visibly fed with a wide range of viral quantification (2 × 10^2^–2.1 × 10^10^ ZIKV cDNA copies) (Fig. 3a).

There was a positive correlation between the ZIKV cDNA copy number recovered from mouse ears and ZIKV cDNA copy number quantified from SGs from the mosquito that fed on each ear (Fig. 3b). In contrast, there was no significant correlation between ZIKV cDNA copy numbers determined for mosquito bodies and corresponding SGs 14 dpi (Spearman’s rho = 0.5052, *P* = 0.0085).

### Viability of ZIKV transmitted by bite to mouse ears

**Fig. 4:**
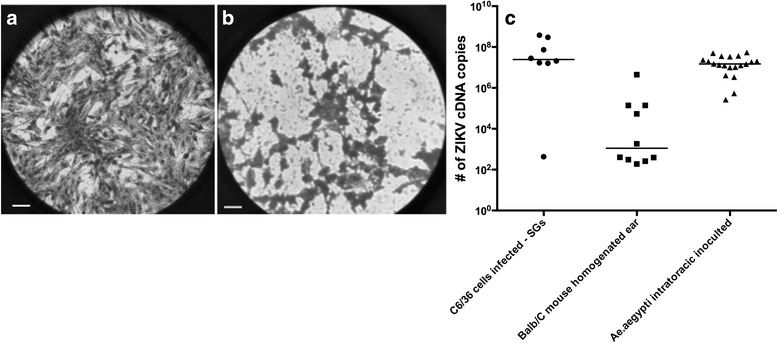
Light microscopy of uninfected and ZIKV-infected C6/36 cell monolayers. The uninfected C6/36 cells are regularly disposed in monolayer covering the entire culture plate (**a**). Distinctly, the C6/36 monolayers that were cultivated following inoculation with ZIKV-infected SGs are showing adjacent cells forming syncytia with empty areas on the culture plate (**b**), structural effect caused by ZIKV invasion of the cells. Numbers of ZIKV cDNA copies in C636 cells infected with SGs, mouse ear homogenates and mosquitoes intrathoracically injected (**c**). *Scale-bars*: 50 μm

Independent assays confirmed the ability of ZIKV transmitted by the bite of *Ae*. *aegypti* to be capable to initiate infections: ZIKV from supernatants of macerated mosquito SGs collected post-bite were able to infect and cause cytopathic effect in C6/36 cells at 4 days of culture (Fig. 4a, b); ZIKV was detected in supernatants from C6/36 cells infected with SGs (from a) by qPCR with viral copy number ranging from 4.3 × 10^2^–3.8 × 10^8^, with a median of 2.45 × 10^7^ cDNA copies; all 20 naïve *Ae*. *aegypti* that were intrathoracically inoculated with infected SGs developed subsequent infection after 14 days. At 14 dpi, the amount of ZIKV in these intrathoracically injected mosquitoes ranged from 2.7 × 10^6^ to 5.4 × 10^7^, with a median of 1.49 × 10^7^ ZIKV cDNA copies. Finally, all cultured homogenates from the macerated ears were ZIKV-positive, with a range of 1.9 × 10^2^–4.4 × 10^5^ and median of 1.15 × 10^3^ ZIKV cDNA copies (Fig. 4c).

## Discussion

Transmission dynamics of ZIKV infection in *Ae*. *aegypti*, or any mosquito vector, depends on infection rates and vector competence. Evaluation of vector competence can be evaluated in three progressive steps: (i) the presence of ZIKV in the vector body demonstrates its ability to invade, replicate and disseminate, and arbovirus discovery in the mosquito salivary glands is required for potential transmission; (ii) the integrity and viability of ZIKV in vector saliva, as verified by cell culture for example, is indicative of the potential of transmission to the vertebrate host by mosquito bites; and (iii) conclusively, the most irrefutable proof of vector competence for ZIKV transmission is detection of viable replicating virus in a vertebrate host following vector bite.

Here, we demonstrated a method that allowed for successful ZIKV transmission by bites of individual ZIKV-infected *Ae*. *aegypti* when they were allowed to probe and feed on BALB/c mouse ears. These experiments were conducted with *Ae*. *aegypti* and ZIKV originating from Brazil, where ZIKV has been circulating efficiently with this mosquito vector [25]. Additionally, mice are a useful laboratory model for ZIKV as other aspects of ZIKV biology and pathogenesis can be explored in this vertebrate [4, 5, 8, 9].

Our experimental model mimics the natural transmission of ZIKV to a vertebrate host by bite in a highly controlled manner; in this example, by individual mosquitoes. In nature, the host is exposed to many mosquito bites, though a single infective bite may be enough to initiate the arboviral infection. Mouse ears are optimal bite/transmission sites as the tissue allows for relatively easy investigation of the infection process. The dermal sheets of the ears are easy to remove and manipulate for pathogen and pathological detection and visualization.

All infected mosquitoes that fed on mice were able to transmit ZIKV. The ZIKV inoculum by *Ae*. *aegypti* bites into mouse tissue ranged from two hundred to several million viral copies and was positively correlated with viral load in the SGs as determined by qPCR. A wide range of infectious titers have previously been observed in this bite model applied to understand sandfly-*Leishmania* transmission [26, 27]. However, the majority of the ZIKV-infected *Ae*. *aegypti* (71%) inoculated relatively large quantities of ZIKV (< 10^6^ copies) by bite in this study. Similarly, experimental bite transmission of West Nile virus to mouse and chick tissues also demonstrated high dose of viral inoculations [17].

In nature, mosquitoes likely become infected with varying virus titers, depending on their feeding behavior and viremia of the infectious host. Similarly host infection and pathology may reflect the amount of virus inoculated at the bite site. There was a positive correlation between the amounts ZIKV recovered in the mouse ears and copy number detected in SG after feeding, but no correlation between ZIKV copy number in SG and mosquito body pairs. It has recently been demonstrated that dengue virus, another flavivius, accumulates and replicates in the SG following dissemination [28]. A similar phenomenon may occur with ZIKV in competent mosquito vectors. These results may suggest that the amounts of ZIKV in SG may be more indicative of productive transmission than that detected in whole mosquito bodies. Unfortunately, most arbovirus surveillance programmes screen pools of whole mosquito bodies, which may provide misleading results for the potential for transmission; however, these programmes, at least, illustrate the presence of virus circulating in vector populations. Recently, it was demonstrated that the concentration of DENV increases post-infection, indicating that the virus can replicate to be accumulate in the SG after the arbovirus dissemination [28]. It is possible that same behavior occurs with the ZIKV, since both are members of the same flavivirus family.

This study conclusively demonstrates that *Ae*. *aegypti* can successfully transmit a replication competent ZIKV to a mouse, in a ZIKV-vertebrate animal infection model that may be further utilized to investigate ZIKV transmission and infection biology. Additionally, replication competent ZIKV was successfully recovered from *Ae*. *aegypti* SGs and was able to infect naïve mosquitoes [23] and induce cytopathic effect in C6/36 cell culture.

## Conclusions

Vector competence requires definitive demonstration of pathogen transmission. For arboviruses, this remains challenging. Additionally, vertebrate animal transmission models to investigate transmission biology or evaluate interventions remain elusive or prohibitively expensive, especially for ZIKV. Here we demonstrate a mosquito-mouse transmission model for ZIKV that may be used for “natural transmission” studies ranging from vector competence to ZIKV transmission biology and immunology. The transmitted dose and infection outcome are impossible to control, since viral copy number may range broadly, as demonstrated in this ZIKV model. However, the methods described here allow evaluation of transmission on potential downstream infection by individual vectors that can be independently assessed for pathogen titer or even saliva chemistry [13]. Additionally, it is important to note that this method replicates transmission by bite, in contrast to recently published ZIKV animal models where the infection is established by syringe inoculation [4, 5, 7–9]. Experiments developed with Semliki Forest virus and Bunyamwera virus demonstrated that the virus transmitted by mosquito bites induced enhanced viral replication at the inoculation site, lead to greater dissemination of the virus, and more lethality, compared with control mice that received the virus by only syringe injection [16]. This is a critical consideration, as it is well demonstrated that transmission by syringe injection, rather than by vector bite, has led to misinterpretation of immune responses to the pathogen and moreover, the efficacy of a killed vaccine [13, 29]. In conclusion, our model illustrates successful transmission of arbovirus ZIKV by infectious mosquito bite. This experimental animal model of ZIKV transmission *via* vector will improve the understanding of the viral pathology as well as initial steps of the cellular immune response at the bite site.

## Abbreviations

dpi: days post-infection
IR: Infection rate
qPCR: quantitative real time PCR
SG: Salivary gland
SG-IR: SG infection rate
TR: Transmission rate
VC: Vectorial capacity
ZIKV: Zika virus
